# Enhanced Virus Resistance in Transgenic Maize Expressing a dsRNA-Specific Endoribonuclease Gene from *E. coli*

**DOI:** 10.1371/journal.pone.0060829

**Published:** 2013-04-09

**Authors:** Xiuling Cao, Yingui Lu, Dianping Di, Zhiyan Zhang, He Liu, Lanzhi Tian, Aihong Zhang, Yanjing Zhang, Lindan Shi, Bihong Guo, Jin Xu, Xifei Duan, Xianbing Wang, Chenggui Han, Hongqin Miao, Jialin Yu, Dawei Li

**Affiliations:** 1 State Key Laboratory of Agro-Biotechnology and MOA Key Laboratory of Soil Microbiology, College of Biological Sciences, China Agricultural University, Beijing, P. R. China; 2 Plant Protection Institute, Hebei Academy of Agricultural and Forestry Sciences, Baoding, P. R. China; Virginia Tech, United States of America

## Abstract

Maize rough dwarf disease (MRDD), caused by several Fijiviruse*s* in the family *Reoviridae*, is a global disease that is responsible for substantial yield losses in maize. Although some maize germplasm have low levels of polygenic resistance to MRDD, highly resistant cultivated varieties are not available for agronomic field production in China. In this work, we have generated transgenic maize lines that constitutively express *rnc*70, a mutant *E. coli* dsRNA-specific endoribonuclease gene. Transgenic lines were propagated and screened under field conditions for 12 generations. During three years of evaluations, two transgenic lines and their progeny were challenged with *Rice black-streaked dwarf virus* (RBSDV), the causal agent of MRDD in China, and these plants exhibited reduced levels of disease severity. In two normal years of MRDD abundance, both lines were more resistant than non-transgenic plants. Even in the most serious MRDD year, six out of seven progeny from one line were resistant, whereas non-transgenic plants were highly susceptible. Molecular approaches in the T12 generation revealed that the *rnc*70 transgene was integrated and expressed stably in transgenic lines. Under artificial conditions permitting heavy virus inoculation, the T12 progeny of two highly resistant lines had a reduced incidence of MRDD and accumulation of RBSDV in infected plants. In addition, we confirmed that the RNC70 protein could bind directly to RBSDV dsRNA *in vitro*. Overall, our data show that RNC70-mediated resistance in transgenic maize can provide efficient protection against dsRNA virus infection.

## Introduction

Maize is a very important global resource for human food and animal fodder, and is also a key bioenergy source. The world production of maize was 844.4 million tonnes in 2010 and maize provides the second highest level of production of all food and agricultural commodities (FAO, http://faostat.fao.org/). In China, maize yields were 208.12 million tonnes in 2012, and were slightly more than rice (204.29 million tonnes), so maize is now our number one grain crop (National Bureau of Statistics of China, Bulletin on the National Grain Output of China in 2012). However several diseases affect maize production, and in China, maize rough dwarf disease (MRDD) complexes contribute substantially to yield losses. The first report of MRDD in China was in 1954 [1], and numerous reports have appeared in different areas since the initial description. In the past few years, disastrous losses caused by MRDD have occurred in most maize growing districts of China. For example, in Shandong province in 2008, the affected area comprised more than 733,000 hm^2^, where 59,100 hm^2^ had to be replanted with other crops and another 16,700 hm^2^ had complete yield losses [1].

*Maize rough dwarf virus* (MRDV), *Mal de Río Cuarto virus* (MRCV) and *Rice black-streaked dwarf virus* (RBSDV), which belong to the genus *Fijivirus* in the family *Reoviridae* [2], each contribute to MRDD syndromes in different maize growing regions. A MRDV association with the MRDD was first noted in Italy [3], and the virus is now known to be distributed broadly in maize growing regions of Europe [4], South America [5] and East Asia [6]–[10], respectively. In China, RBSDV was first found in MRDD maize plants in 2000 [9], and subsequent studies have confirmed that the etiological agent of MRDD in China is RBSDV, rather than MRDV [8], [11], [12]. The RBSDV genome consists of 10 double-stranded RNA (dsRNA) segments [2], and the virus is obligately transmitted by the small brown planthopper (*Laodelphax striatellus*), in which it multiplies. RBSDV can infect several cereal crops including rice, maize, wheat and sorghum [13]–[16], so a significant disease reservoir exists in China.

**Figure 1:**
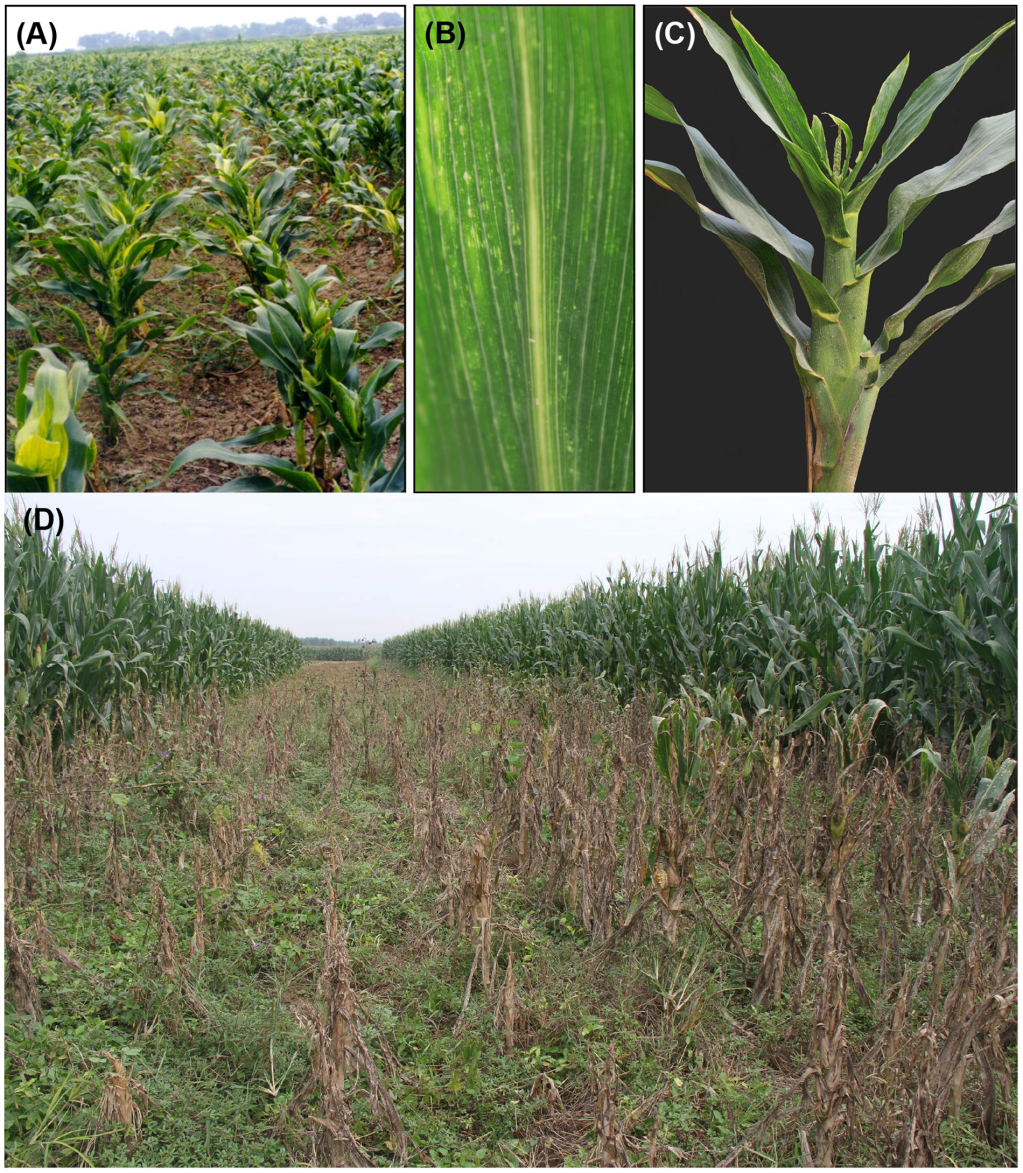
Symptoms of maize rough dwarf disease (MRDD) caused by *Rice black-streaked dwarf virus* (RBSDV) in the field. (**A**) MRDD symptoms on maize heavily infected with RBSDV under natural field conditions. (**B and C**) Lower surfaces of a RBSDV infected leaf showing dark leaf greening and vein clearing with small white enations and plant dwarfing. **Note:** Plants were photographed at 4 weeks after feeding access by viruliferous planthoppers. (**D**) Effects of different planting dates on MRDD incidence. The center plot shows MRDD in heavily infected RBSDV maize that had been planted in northern China at the end of May, 2011 before harvest of surrounding wheat fields. Under these conditions, high concentrations of planthoppers migrate from maturing wheat to emerging maize seedlings and transmit RBSDV. The vigorous plants on each side of the RBSDV-infected plants were planted on June 18, 2011 after wheat in surrounding was harvested and most of the planthoppers had dispersed. The infected plants failed to develop tassels and had very small cobs with greatly reduced seed set, and often died. Plants were photographed on Sep. 7, 2011. **Note:** This area of China is in a wheat-maize rotation region and maize planted before wheat harvesting generally develops very high levels of MRDD because planthoppers preferentially move from mature wheat to new maize seedlings.

RBSDV-infected maize exhibits severe growth abnormalities, including plant dwarfing, dark leaf greening and a vein clearing that often results in white streaks with small white enations on the lower surfaces of leaves and sheaths. In the most severe cases, infected plants fail to set seed or produce ears [17], [18] (Figure 1). Maize germplasm harbors different resistance responses to MRDD under natural infection [19]–[23]. These studies have shown that maize resistance to RBSDV is a quantitative characteristic controlled by polygenes, each of which has minor effects [24]–[26]. So far, a major dominant gene encoding RBSDV resistant has not been identified genetically. Moreover, highly resistant cultivated maize varieties of any sort that can be used to control MRDD in the field are not available in the Chinese elite heterotic groups [1], [21], [23], [27]. Thus, the result is that all cultivated maize varieties in agricultural production are rated as either susceptible or highly susceptible. Because of these problems, it has not been possible to breed new resistant lines by traditional breeding. Therefore, current control measures rely on using pesticides to kill vector insects, or altering the sowing time to improve field management [26], [28].

However, another environmentally safe and cost-effective option for reducing crop losses is resistance mediated by transgenes. Since the pathogen-derived resistance (PDR) concept was first proposed [29], various transgenic approaches based on viral genes and sequences have been applied to many plant species [30]–[33]. Nowadays, three-quarters of virus-resistant transgenic plants express viral coat protein (CP) genes, and the remaining transgenic plants contain a large variety of other viral coding sequences and non-coding sequences to confer resistance [32], [33]. Nevertheless, these approaches have potential problems, because the resistance generated is generally effective against only one kind of virus or closely related viruses. In addition, viral RNA derived resistance could possibly lead to selection of mutant viruses that could evade transgenic resistance targeting. Apart from the multitude of PDR approaches, a number of alternative strategies for antiviral resistance in transgenic plants using various genetic resources have been explored over the past two decades [32]–[34]. Amongst these, expression or induced expression of dsRNA-specific ribonucleases has been shown to be successful [35]–[41]. Sano *et al*. generated transgenic potato lines expressing the yeast-derived dsRNA-specific ribonuclease gene (*pac1*), and found that transgenic potato and progeny potato tubers could suppress *Potato spindle tuber viroid* (PSTVd) accumulation during infection and that the protein PAC1 could digest PSTVd *in vitro* [38]. Ishida *et al.* came to the same conclusion with *pac1* transgenic tobacco and chrysanthemum, which also exhibited resistance to viroid and virus infections [39]. Transgenic tobacco harboring the *E. coli rnc* gene encoding an RNase III endoribonuclease and a mutant gene, *rnc*70, were resistant to infection of several disparate RNA plant viruses with divided genomes, but not against viruses with a single-stranded RNA genomes [37]. In addition, wheat transformed with *rnc*70 exhibited high levels of resistance to *Barley stripe mosaic virus* (BSMV) infection [40].

Ribonuclease III (RNase III) represents a highly conserved family of dsRNA-specific endoribonucleases that have important roles in RNA processing, post-transcriptional gene expression control and other processes initiated by dsRNA in both prokaryotes and eukaryotes [42]–[45]. RNase III encoded by the *rnc* gene in *E. coli* has the simplest protein structure in the family and also is a well-studied family member. RNC70 is an RNase III (E117K) mutant that has been proven to bind but not cleave dsRNAs *in vitro* [46]–[48]. In this study, we demonstrate that two RNC70 transgenic maize lines recovered after more than ten generations of self-crossing and selection have high levels of resistance to MRDD caused by RBSDV. This is the first report of transgenic maize lines with effective resistance to RBSDV in the field.

## Results

### Generation of Transgenic Maize Lines Carrying *rnc* and *rnc*70

**Figure 2:**
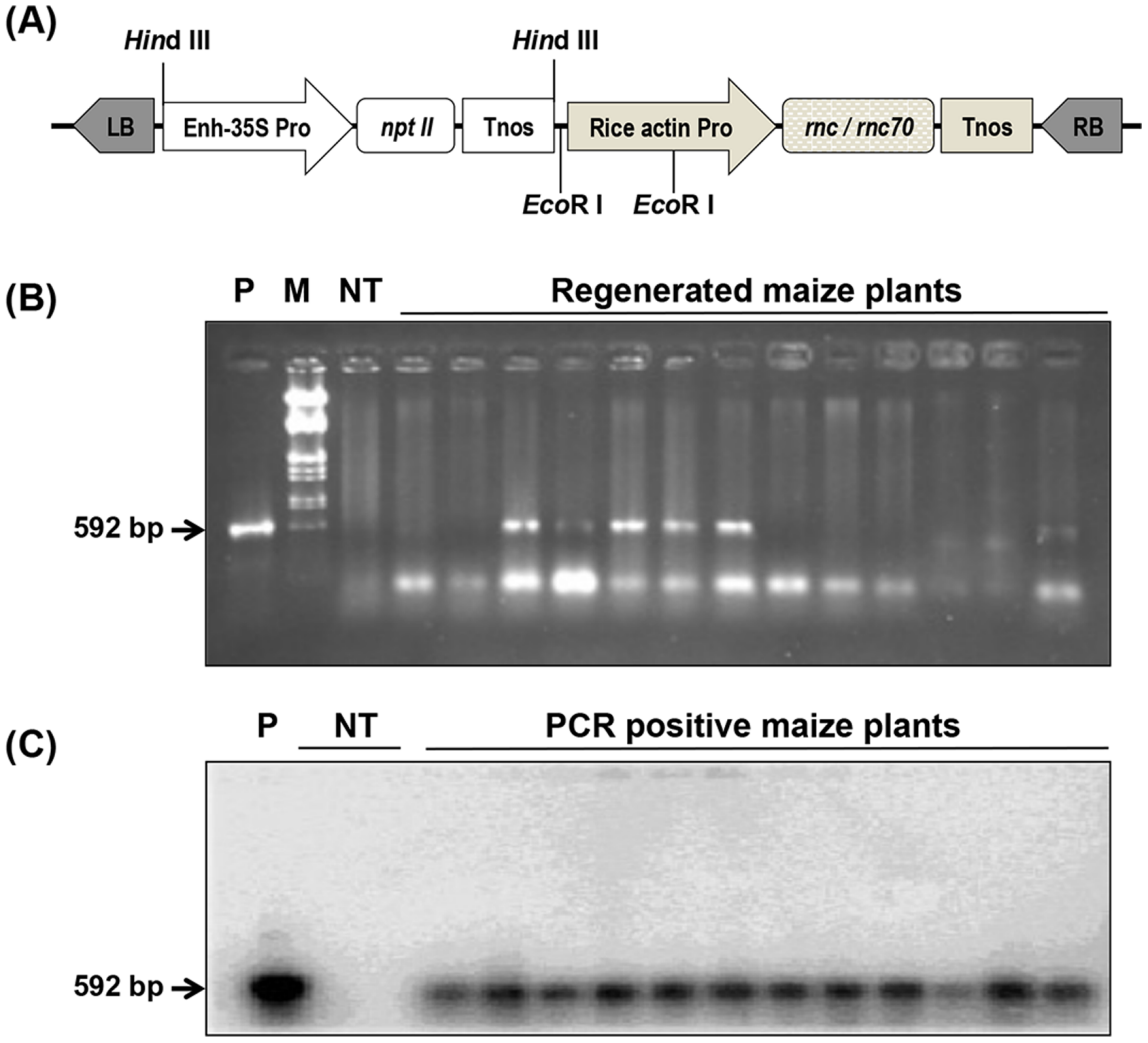
Construction of plant expression vectors and molecular detection of transgenes. (**A**) Schematic diagram of the plant expression vectors pAMM2024 and pAMM2025, which are derivatives of vector pCB301 [68]. The bacterial RNase III *rnc* (pAMM2024) and the E117K (*rnc*70) mutant (pAMM2025) genes were inserted between the rice *Actin* promoter and the nopaline synthase 3′ terminator (Tnos). Enh-35S, enhanced 35S promoter with a duplicated enhancer; NPT II, neomycin phosphotransferase II gene; RB, right border; LB, left border. (**B and C**) PCR and PCR-southern blot detection of transgenes. T0 maize plants were detected by PCR first (B), and the 592 bp PCR products from positive plants were confirmed by southern blot using the *rnc*-specific probe (C). P: The pAMM2025 plasmid was used as a positive control; M: λDNA/*Eco*RΙ+*Hind* III markers; NT: non-transgenic plants.

RBSDV genomic RNA is composed of 10 double-stranded RNAs that might be potential substrates for RNase III. To identify anti-RBSDV activity of RNase III, two maize inbred lines (Z3 and Z31 genotype), were transformed with pAMM2024 or pAMM2025, in which the full-length *E. coli* RNase III cDNA (*rnc* gene) and the E117K mutant (*rnc*70) were inserted downstream of the rice actin promoter respectively (Figure 2A). Maize genotypes have different responses to T-DNA delivery [49], [50], and we noted that the Z3 and Z31 genotypes were transformed at efficiencies of 0.00% and 2.33% with pAMM2024, and 0.65% and 1.62% with pAMM2025 respectively (Table 1). In all, sixty-four T0 transgenic lines were generated and confirmed by PCR-southern blot assays (Figure 2B and C). Among the T0 lines, twenty-five lines contained the wild-type *rnc* gene, and thirty-nine lines were transformed with the *rnc*70 mutant (Table 1). Compared with the *rnc*70 lines, transgenic maize expressing the wild-type *rnc* gene exhibited anomalous phenotypes, including stunted and slow growth, leading to no progeny seeds in the T1 generation, these results are consistent with previous studies in transgenic tobacco [37].

**Table 1:** PCR detection of regenerated maize carrying the *rnc* and mutant (*rnc*70) transgenes.

Plasmid	Transgenic recipient	Numbers of immature embryosused for transformation	Numbers of regenerated plants	Numbers of positive plantby PCR-Southern	Positive rate* (%)
pAMM2024	Z3	1300	3	0	0.00
pAMM2024	Z31	1073	48	25	2.33
pAMM2025	Z3	2320	26	15	0.65
pAMM2025	Z31	1480	47	24	1.62

* Numbers of positive plant by PCR-Southern/numbers of immature embryos for transformation ×100%.

### Propagation and Screening of Transgenic Maize Lines in the Field

**Figure 3:**
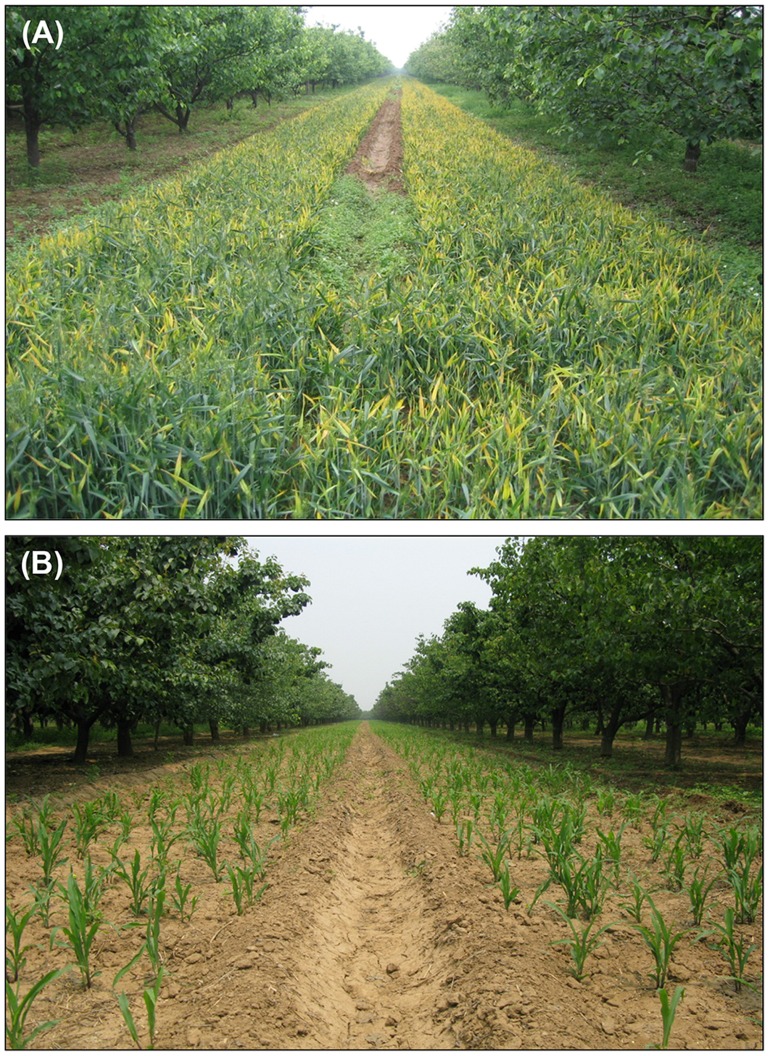
Screening fields with an unusually high disease incidence in the area. The screening field depicted above had serious infections of RBSDV in the surrounding wheat crops and in weeds. The screening fields were chosen because they provided wheat and weed habitats sufficient to permit growth of large populations of the small brown planthopper before transgenic maize was planted in the spring (A). Consequently, high densities RBSDV-infected planthoppers migrated into the fields after planting of the test plants in the summer (B). After maize was planted, high levels of infection were observed in the control non-transgenic plants and in the highly susceptible maize variety, and high densities of RBSDV-infected planthoppers were detected in all plants. To mediate bio-safety protection, an isolation belt consisting of surrounding pear trees was considered in the initial design to provide protection against pollen flow and spread of transgenic maize [74]. The photographs were taken on May 15 (A) and June 12 (B), 2010 respectively.

To obtain transgenic maize lines with high levels of resistance to MRDD, 101 T2 PCR-positive transgenic maize lines were challenged in the field. All of the transgenic maize lines were maintained entirely by self-pollination within each transgenic line in every generation. Because RBSDV, the cause of MRDD in China, is transmitted by small brown planthoppers (*Laodelphax striatellus*), the density of the planthoppers carrying virus is an important factor affecting MRDD field incidence and spread. In addition, temperature and humidity also contribute to disease outbreaks [21]. Keeping these factors in mind, we screened several fields in which MRDD exhibited an unusually high disease incidence (Figure 3) to evaluate resistance elicited by the transgenic maize lines. To further maximize infection efficiencies, the transgenic lines were planted at specific times to correlate the most susceptible periods of maize development with the maximum migration of planthoppers into the field plots [21].

**Figure 4:**
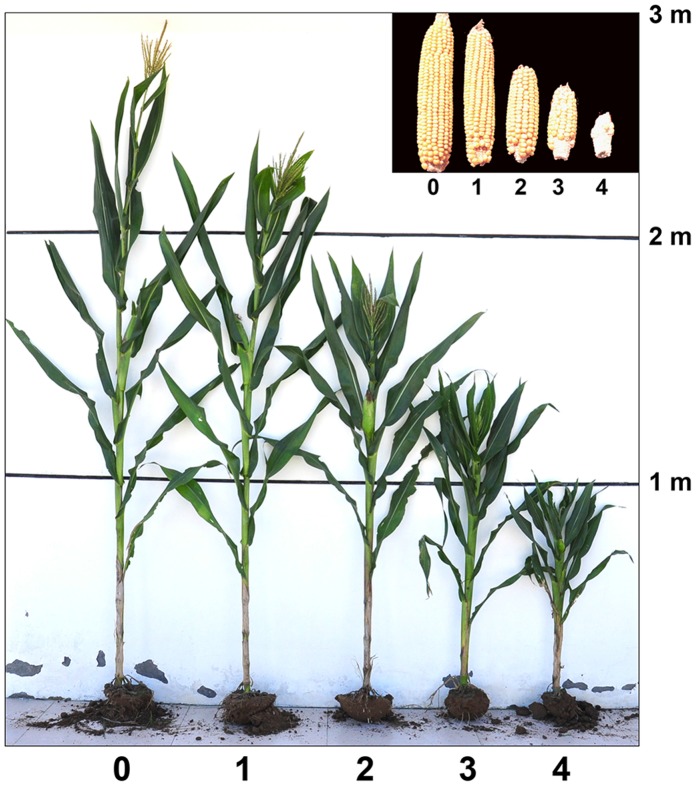
MRDD symptoms scored on a 0 to 4 disease scale. Disease scores were assessed as follows: 0 (no visible symptoms), 1 (4/5 of normal healthy plant height, with white streaks throughout the veins of upper leaves), 2 (2/3 of healthy plant height, with visible dwarfing, combined with dark greening and white streak symptoms throughout the plant), 3 (more extreme dwarfing; ∼1/2 of healthy plant height, delayed tassels that failed to develop pollen, and very small cobs; and 4 (1/3 of healthy plant height, with no cobs or dwarf cobs containing only a few seed, or plant death). The inset shows cobs taken from plants with scores of 0 to 4, respectively.

Disease indices (DI) and MRDD symptoms were evaluated during the milk stage of maize seed development. Disease scores were defined as follows: 0 (no visible symptoms), 1 (4/5 of normal healthy plant height, with white streaks throughout the veins of upper leaves), 2 (2/3 of healthy plant height, with visible dwarfing, combined with dark greening and white streak symptoms throughout the plant), 3 (more extreme dwarfing; ∼1/2 of healthy plant height, delayed tassels that failed to develop pollen, and very small cobs; and 4 (1/3 of healthy plant height, with no cobs or dwarf cobs containing only a few seed, or plant death) (Figure 4). Based on the disease score and the susceptibility rate, DI scores were calculated and analysed [51] (Table 2). According to the DI, the maize lines were divided into highly resistant (0.0–10.0), resistant (10.1–20.0), susceptible (20.1–40.0) and highly susceptible (40.1–100.0). The most highly resistant lines with positive genomic PCR bands were selected for the next round of evaluation.

**Table 2:** Numbers of MRDD resistant and susceptible transgenic progeny in the field.

Grade	T2 (Summer, 2006)	T4 (Summer, 2007)	T6 (Summer, 2008)
Highly resistant	12	57	27
Resistant	37	97	21
Susceptible	40	105	9
Highly susceptible	12	14	1
Total number	101	285	57

Resistance was assessed according to the disease index: Highly resistant = 0.0–10.0; Resistant = 10.1–20.0; Susceptible = 20.1–40.0; Highly Susceptible = 40.1–100.0.

See Figure 4 for disease scores. The disease indices were calculated as described by Powell *et al.* (1971) [51]; [Disease index (%) = [(∑ plants number × the corresponding disease score)/(the highest disease score × the total plant number)] ×100].

As shown in Table 2, 11.9%, 20.0% and 47.4% of the T2, T4 and T6 generation, respectively were highly resistant to MRDD. In these experiments, transgenic maize was planted in the winter in tropical field plots in southern China and in northern areas in the summer to obtain two generations of seed per year. The resistance of the T3 and T5 generations was not evaluated during the winter in tropical field plots because of the low populations of *L. striatellus* carrying RBSDV in the field and the lack of optimal temperatures for RBSDV infection. In the T6 generation, all of the highly resistant transgenic maize lines were progeny of the ND60 and ND67 T2 generation transgenic lines, both of which had been transformed Z31 inbred line with *rnc*70. These results suggest that the *rnc*70 resistance in the transgenic lines was durable and had a selective advantage over 5 generations (T2 to T6) of seed increase during the field conditions present from 2006 to 2008.

The systematic PCR analyses conducted to detect the transgene in the T6 generation revealed that all of the samples were positive for *rnc*70. Moreover, only a single *rnc*70 band was detected in southern blot analyses of the samples, indicating that *rnc*70 had integrated only as a single copy gene. In addition, the ND60 and ND67 progeny exhibited no obvious changes from the recipient inbred line (Z31) in either yield potential or other agricultural traits.

### The ND60 and ND67 Transgenic Maize Lines Exhibit High Field Resistance to MRDD

To investigate the performance of the highly resistant transgenic maize lines under different disease stress conditions, we conducted field trials of the ND60 and ND67 T6, T8 and T10 generations in the summers of 2008, 2009 and 2010 (Table 3). Because the outbreak of plant viruses induced diseases is affected by numerous biotic and abiotic factors, we investigated MRDD resistance of transgenic maize lines in several experimental field plots. To provide a control for disease stress induced by RBSDV in the field, we compared the transgenic lines with the cultivated maize variety, Ye107, which is high susceptible to RBSDV.

**Table 3:** Field evaluations of ND60 and ND67 transgenic *rnc*70 and control lines for MRDD resistance in 2008, 2009 and 2010.

Year	Transgenic lines	Total plants	Disease score					Susceptibility rate (%)	Disease index (%)
			0	1	2	3	4		
2008 (T6)	ND67-1-3-1-1	19	18	0	1	0	0	5.3	2.6^a^
	ND67-1-3-1-2	18	18	0	0	0	0	0.0	0.0^a^
	ND67-1-3-2-1	19	18	0	1	0	0	5.3	2.6^a^
	ND67-1-3-2-2	20	20	0	0	0	0	0.0	0.0^a^
	ND67-1-3-5-4	20	20	0	0	0	0	0.0	0.0^a^
	ND67-1-3-5-5	13	13	0	0	0	0	0.0	0.0^a^
	ND60-2-5-4-1	20	19	0	1	0	0	5.0	2.5^a^
	ND60-2-5-4-2	14	14	0	0	0	0	0.0	0.0^a^
	Z31*	40	34	2	1	2	1	15.0	8.8^a^
	Ye107 **	42	20	0	0	0	22	52.4	52.4^b^
2009 (T8)	ND67-1-3-1-1	15	15	0	0	0	0	0.0	0.0^a^
	ND67-1-3-1-2	17	17	0	0	0	0	0.0	0.0^a^
	ND67-1-3-2-1	29	29	0	0	0	0	0.0	0.0^a^
	ND67-1-3-2-2	13	13	0	0	0	0	0.0	0.0^a^
	ND67-1-3-5-4	27	24	2	1	0	0	11.1	3.7^b^
	ND67-1-3-5-5	84	81	3	0	0	0	3.6	0.9^a^
	ND60-2-5-4-1	50	44	6	0	0	0	12.0	3.0^b^
	ND60-2-5-4-2	49	43	4	2	0	0	12.2	4.1^b^
	Z31*	51	40	2	4	5	0	21.6	12.3^b^
	Ye107**	60	30	9	3	9	9	50.0	32.5^c^
2010 (T10)	ND67-1-3-1-1	42	15	4	6	10	7	64.3	32.8^cde^
	ND67-1-3-1-2	158	61	8	23	47	19	61.4	42.9^cd^
	ND67-1-3-2-1	187	67	28	33	39	20	64.2	39.0^bc^
	ND67-1-3-2-2	77	48	7	4	10	8	37.7	24.3^a^
	ND67-1-3-5-4	114	54	6	22	24	8	52.6	35.5^abc^
	ND67-1-3-5-5	146	61	30	22	22	11	58.2	32.8^ab^
	ND60-2-5-4-1	139	36	6	26	44	27	74.1	53.6^ef^
	ND60-2-5-4-2	137	34	4	27	57	15	75.2	52.7^def^
	Z31*	135	37	14	10	27	47	72.6	56.2^f^
	Ye107**	141	4	7	2	9	119	97.2	91.2^g^

Measurements of disease score and disease indices are as in Figure 4 and Table 2. Values with different superscripts in the disease index column are significantly different (P≤0.05), as assessed by Duncan’s multiple range test.

* Non-transgenic plants;

** highly susceptible MRDD variety as a control.

In 2008 and 2009, Ye107 had normal susceptibility rates (52.4% and 50.0%) and disease indices (52.4 and 32.5), indicating that RBSDV field stress was relative low. Nonetheless, both of the T6 and T8 ND60 lines and ND67 lines exhibited high MRDD resistance (DI 0.0–2.6, 0.0–4.1). In 2010, MRDD was prevalent in the susceptible Ye107 control variety (97.2% and DI of 91.2), and non-transgenic Z31 plants also exhibited high susceptibility rates (72.6% and DI of 56.2). In contrast, six out of seven transgenic ND67 lines in generation 10 (T10) were resistant, but two of the ND60 progeny lines that were resistant in 2008 and 2009 exhibited the similar levels of susceptibility as the non-transgenic Z31 plants (Table 3).

**Figure 5:**
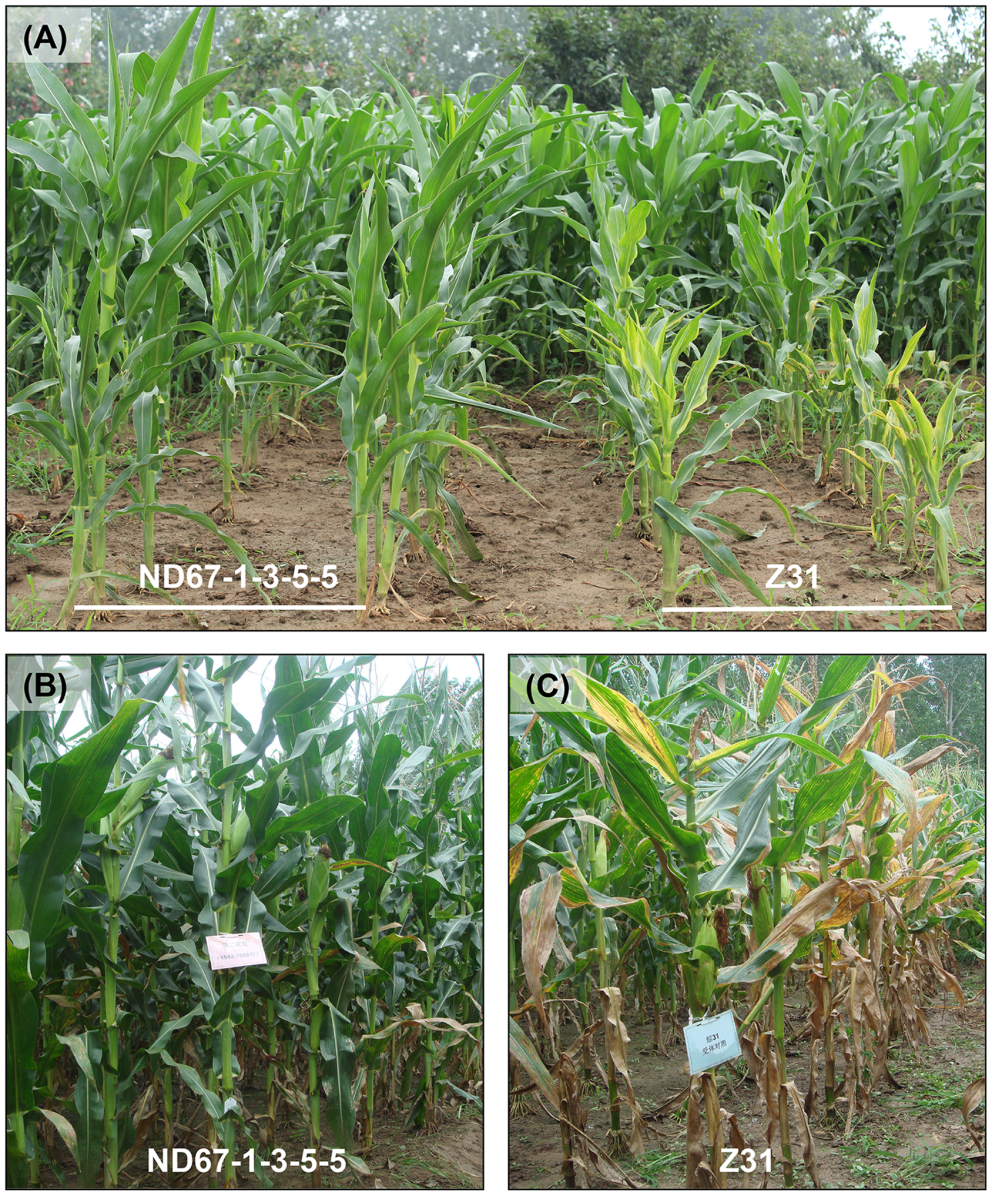
Field showing MRDD in transgenic maize plants with (*rnc*70) resistance to RBSDV infection. (**A**) Comparison of immature transgenic plants (ND67-1-3-5-5) that are highly resistant to RBSDV compared to non-transgenic Z31 plants that are susceptible to RBSDV. The plants were photographed at 9 weeks after sowing. **Note:** The plants in the background are part of a protective buffer designed to ensure the biosafety of transgenic plants. (**B and C**) Appearance of mature transgenic plants (ND67-1-3-5-5) and non-transgenic Z31 maize near harvest time. Note the differences in sizes and appearances of the transgenic and non-transgenic plants. ND67-1-3-5-5 leaves maintained a normal green color, but leaves of the non-transgenic Z31 stunted plants developed strong yellow chlorotic regions.

In each of the three stress years tested, all T6, T8 and T10 ND67 transgenic lines maintained stable levels of high MRDD resistance compared with the non-transgenic plants (Figure 5), and, except for 2010, most ND60 transgenic lines were also remarkably resistant to disease development, whereas both lines had better performance than the non-transgenic Z31 line (Table 3). Among the progeny lines ND60 (ND60-2-5-4-1) and ND67 (ND67-1-3-5-5) consistently exhibited outstanding performance. Overall, these results provide evidence that the *rnc*70 gene was stably integrated into the transformed lines and was maintained throughout several generations of variable disease and field selection conditions, which also strongly suggest that introduction of *rnc*70 provides resistance to RBSDV in the field.

### RBSDV Resistance Following Challenge with a High Population of Virulent Vectors

As described above, variations in the major biotic and abiotic factors affecting the occurrence of RBSDV in the field during 2008, 2009 and 2010 created difficulties in evaluating in transgenic resistance to MRDD. To more precisely control the inoculation conditions and virus titer, we developed a modified procedure, in which small brown planthoppers (*L. striatellus*) were used to inoculate transgenic and control maize lines under laboratory conditions. In these experiments, ND67-1-3-5-5, ND60-2-5-4-1 T12 lines and Z31 recovered in 2011 were challenged with virulent small brown planthoppers by allowing a 3 day inoculation access period (IAP) on plants to be tested. Each maize line had three different replicates, and the experiments were carried out twice. Under the IAP conditions, all tested maize lines developed symptoms similar to those observed in the 2010 field tests (Table 4). The non-transgenic Z31 plants and the transgenic ND60-2-5-4-1 lines developed an intense MRDD phenotype (DI was 74.0–92.5 and 90.3–96.3 respectively) that was much more pronounced than that of the transgenic ND67-1-3-5-5 plants (DI was 49.0–67.9).

**Table 4:** MRDD symptoms on ND60-2-5-4-1 and ND67-1-3-5-5 T12 transgenic lines at 2 weeks after access feeding by viruliferous planthoppers.

	Maize lines		Total plants	Disease score					Susceptibilityrate (%)	Disease index (%)
				0	1	2	3	4		
Exp. 1	ND67-1-3-5-5	I	21	0	3	3	12	3	100.0	67.9^b^
		II	24	2	4	12	5	1	91.7	49.0^a^
		III	18	2	2	4	6	4	88.9	61.1^ab^
	ND60-2-5-4-1	I	22	0	0	2	3	17	100.0	92.1^c^
		II	18	0	1	0	4	13	100.0	90.3^c^
		III	20	0	0	0	3	17	100.0	96.3^c^
	Z31*		20	0	2	0	0	18	100.0	92.5^c^
Exp. 2	ND67-1-3-5-5	I	22	0	6	6	6	4	100.0	59.1^a^
		II	24	1	9	4	8	2	95.8	51.0^a^
		III	20	4	2	5	8	1	80.0	50.0^a^
	ND60-2-5-4-1	I	24	2	4	1	3	14	91.7	74.0^b^
		II	24	0	2	0	0	22	100.0	93.8^b^
		III	17	0	1	1	1	14	100.0	91.2^b^
	Z31*		19	0	3	0	1	15	100.0	86.8^b^

Measurements of disease score and disease indices are as described in Figure 4 and Table 2. The numbers I, II and III represent three independent progeny selected from lines tested in the previous generations. Values with different superscripts in the disease index column are significantly different (P≤0.05) by Duncan’s multiple range test.

* Non-transgenic plants.

**Figure 6:**
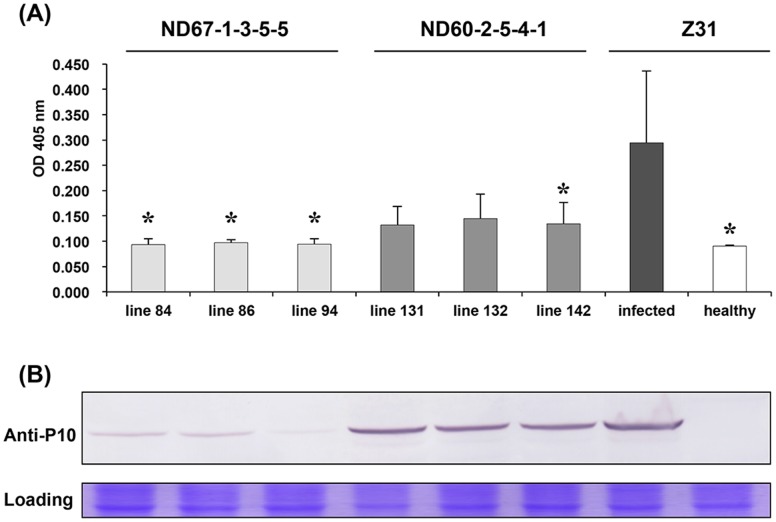
ELISA and western blotting to assess the resistance of transgenic maize under artificial inoculation conditions. (**A**) ELISA of three independent lines derived from ND67-1-3-5-5 and ND60-2-5-4-1 transformations, compared with healthy and infected non-transgenic Z31 controls. The OD_450_ values illustrate the accumulation of RBSDV and the data presented are the means from three independent samples of each line with the standard deviation shown as error bars. The transgenic lines were compared with infected non-transgenic lines, and differences within each line from infected non-transgenic lines were tested for significance by Student’s t-tests (*P<0.05). (**B**) Western blot analysis of the same samples as those used for the ELISA. Protein was isolated from the infected transgenic and non-transgenic maize leaves. Antibodies specific for the RBSDV P10 protein (anti-P10) [73] was used for RBSDV detection, and healthy Z31 was used as the negative control. The bottom panel shows a protein loading control with Coomassie blue staining.

We also conducted ELISA and western blots assays to quantitate the accumulation of RBSDV in diseased plants (Figure 6A and B). Consistent with the observed MRDD symptoms, the accumulation of RBSDV in the ND67-1-3-5-5 line was lower than in the ND60-2-5-4-1 and non-transgenic maize lines. However, compared to the non-transgenic Z31 plants, infected ND60-2-5-4-1 had a substantially lower accumulation of RBSDV virus in spite of developing similar severe disease symptoms. This result suggests that expression of *rnc*70 in transgenic maize not only depresses the incidence of MRDD in transgenic plants exhibiting high levels of protection, but also reduces the accumulation of RBSDV in plants that show lower levels of MRDD losses. Therefore, even modest levels of RBSDV control by *rnc*70 could contribute to lower MRDD field disease indices by reducing virus acquisition during vector feeding.

### RNC70 Interacts Directly with the RBSDV dsRNA Genome

**Figure 7:**
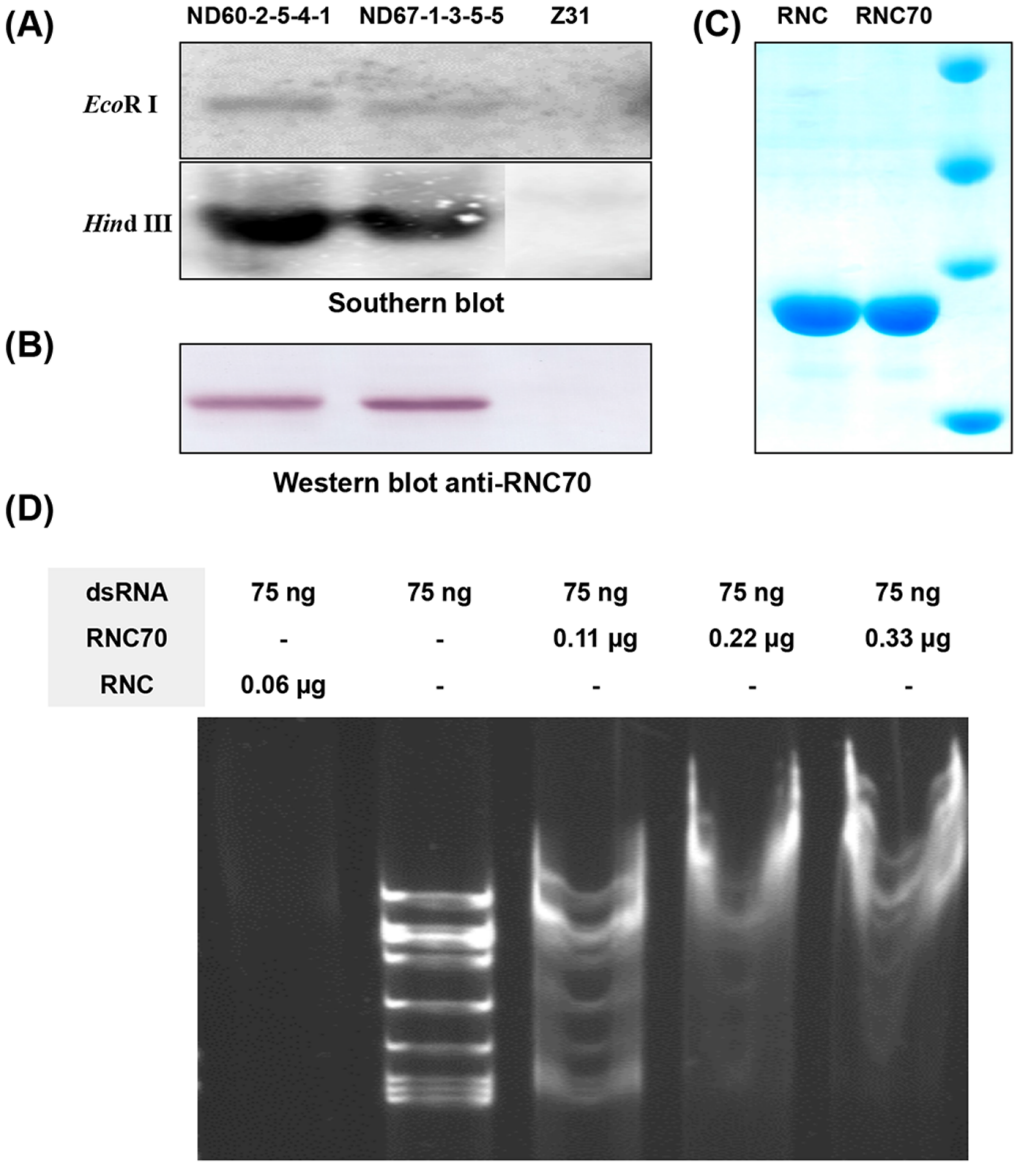
Molecular detection of transgene expression in T12 transgenic maize and assays for RNC70 binding to RBSDV dsRNA. (**A and B**) The *rnc*70 gene was stably integrated into the genomes of transgenic maize, as shown by southern blot analysis with an *rnc*70 probe, excised by *Eco*R I and *Hin*d III respectively (A). Expression of the RNC70 protein in the transgenic maize was confirmed by western blot analysis using anti-RNC70 antibodies (B). (**C and D**) In the binding assays, the RNC70 and RNC proteins were expressed and purified in *E. coli* (C). RBSDV dsRNA (75 ng) was incubated with RNC (0.06 µg) and RNC70 (0.11, 0.22 or 0.33 µg) for 30 min at 37°C and separated on a 5% native polyacrylamide gel. In contrast to RNC, in which RBSDV dsRNA was completely cleaved, the rates of RBSDV dsRNA migration were progressively reduced after incubation with increasing amounts of the RNC70 protein (D).

Given that RNC is known to cleave dsRNA, and that RNC70 can bind dsRNA without cleavage, we predicted that transgenic RNC70 plants might mediate resistance by binding to RBSDV genomic dsRNA. To evaluate this notion, southern and western blot assays were carried out with extracts from the transgenic maize lines. The southern blot results confirmed that lines ND67-1-3-5-5 and ND60-2-5-4-1 contain a single copy of the *rnc*70 gene and express the full length RNC70 protein (Figure 7A and B). Then, his-tagged RNC and RNC70 proteins were expressed in *E. coli* and purified by metal affinity chromatography (Figure 7C) for *in vitro* binding assays to determine whether the recombinant proteins could bind and/or cleave dsRNA from the RBSDV genome. His-tagged RNC and RNC70 proteins were incubated with RBSDV genomic dsRNA, and then separated on 5% native PAGE gels. As shown in Figure 7D, the dsRNA genome of RBSDV was completely cleaved by RNC. In contrast, RNC70 reduced the migration rates of the dsRNAs in the gel and failed cleave the dsRNAs. To our knowledge, this is the first report to show that the E117K mutant (RNC70) participates in binding viral dsRNA but is deficient in cleavage, and this provides some evidence to explain the mechanism of resistance.

## Discussion

In this work, we have generated several transgenic maize lines expressing RNC70. Among the lines, ND67-1-3-5-5 was highly resistance in most challenge tests, and was more resistant than the other tested transgenic line (ND60-2-5-4-1) or non-transgenic plants (Tables 3 and 4). It should be noted that high levels of resistance was observed under field conditions where a wide range of complex biotic and abiotic factors could affect plant disease resistance. Although screening for transgenic progeny resistant to pathogen infections is normally performed under artificial inoculation conditions, screening in the field, as undertaken in our study, provides an efficient and economical means for evaluating virus-resistant transgenic plants [52]–[57]. Interestingly, although the stable RNC70 expression levels in T12 transgenic plants were similar (Figure 7B), resistance against RBSDV in the ND67 lines was higher than those of the ND60 lines, both in the field and under artificial inoculation conditions (Tables 3 and 4). These differences could be due to copy number, insertions at positions that affect transcription, different levels of transgene silencing and epigenetic effects, each of which could cause variations in the levels of protein expression and result in reduced yields [58]–[60].

In addition to causing a major disease in maize, RBSDV can infect wheat, rice and sorghum, where it can also cause serious losses [13]–[16]. RBSDV invades rice and causes rice black streaked dwarf disease, which is one of most serious viral diseases in rice in China. Wheat and sorghum, infected with RBSDV also exhibit dwarfing syndromes. RBSDV is obligately transmitted to each of these cereal hosts by the small brown planthopper (*L. striatellus*) [61]. Because the major hosts of the small brown planthopper are very important cereal crops that are grown throughout China, an RBSDV epidemic in one crop could easily have effects on contiguous crops and result in yield losses in all of the crops. Continuous cropping or mixed cropping, as well as the presence of weed hosts of the small brown planthopper throughout the entire year also leads to persistent cycles of infection. For example, the wheat-maize rotation areas of northern China are especially hard-hit by MRDD, and the main reason for this is planthopper transmission of the virus from wheat to maize seedlings [1]. Our results suggest that expression of *rnc*70 in transgenic maize could not only reduce MRDD losses, but could also affect RBSDV reservoirs in maize field, thus decreasing the amount of viruliferous planthoppers migrated subsequently into wheat, rice and other crops.

In 2008, a new *Fijivirus* species, *Southern rice black streaked dwarf virus* (SRBSDV), closely related to RBSDV, was reported in a rice field in southern China [62], [63]. Since then, SRBSDV has been identified in rice fields in other provinces in China and in Vietnam, and has even been diagnosed in a maize field in northern China [62]–[66]. If SRBSDV spreads, it will be very difficult to control, because the insect vector, the white-backed planthopper (*Sogatella furcifera*), is a wide-spread pest that has an extensive migration range in China and many other Asian countries, and hence can be expected to effectively transmit the virus from rice to maize crops [63], [67]. We have verified that use of RNase III-based transgenic maize is a viable strategy for RBSDV control that likely can be applied to other transgenic crops to prevent virus diseases, particularly those caused by dsRNA viruses. For example, previous studies have shown that *rnc*70 transgenic tobacco plants are resistant to infection by several disparate RNA plant viruses with divided genomes [37], and wheat transformed with *rnc*70 exhibits resistance to BSMV infection [40].

In summary, our study shows that transgenic maize plants harboring *rnc*70 exhibit resistance to RBSDV and suggests that the transgene likely will be effective against other phytoreoviruses. Moreover, wide spread utilization of *rnc*70 resistance should reduce transmissibility of RBSDV in the field and provide protection to RBSDV host crops in the same area. Hence, in addition to wheat and maize, we expect that *rnc*70 resistance will be applicable to rice, sorghum and other field crops.

## Materials and Methods

### Construction of *rnc* Gene and its Mutant (*rnc*70) Expression Vector

The expression vectors, pAMM2024 and pAMM2025, a gift from professor Amitava Mitra (Department of Plant Pathology, University of Nebraska-Lincoln), contain the gene *rnc* from *E. coli* (GenBank accession number X02946) and its E117K mutant (*rnc*70), respectively. The backbone of those two plasmids is pCB301, which is a mini binary transgenic vector that contains the broad-host-range RK2 replication origin *nptII* for kanamycin resistance and the T-DNA border sequences with the multiple cloning site (MCS) polylinker from pBlueScript II [68]. The 681 bp ORFs of *rnc* and *rnc*70 were inserted into MCS sites between the rice *Actin* 1 promoter and nopaline synthase (*Nos*) transcription terminator (Figure 2A). The binary expression plasmids containing the *rnc* or *rnc*70 genes were transferred to *Agrobacterium tumefaciens* strain LBA4404 for maize transformation.

### Maize Transformation and Identification

The maize inbred lines Z3 and Z31 belong to one of six Chinese elite heterotic groups. These two lines have been used for development of several hybrid progeny lines grown throughout China [69] and exhibit high genetic transformation efficiency and are widely used as transgene recipients in China [70]. Transformation of Z3 and Z31 was achieved through an *Agrobacterium*-mediated approach as previously described [71]. Briefly, immature zygotic embryos of the inbred maize lines were aseptically dissected from ears harvested 10 to 13 days post pollination and transformed in four different combinations using the expression vectors (pAMM2024 and pAMM2025) and two inbred lines (Z3 and Z31). T0 plants were regenerated in tissue culture and selected on 10 mg/L kanamycin media. The *rnc* and *rnc*70 genes were identified in transformed plants by PCR with the pRN3-3 (5′-CAACGGAAGCTGGGCTACAC-3′) primer corresponding to 28–47 nt of the *rnc* gene and the reverse pRN3-4 primer (5′-TTGAACCTGTGCCAACCACC-3′) complementary to 619–600 nt of the *rnc* gene. The PCR products from the transgenic plant genome DNA were transferred to nylon membranes, followed by hybridization with the ^32^P-labeled probe from the *rnc*70 gene to provide PCR-Southern blots.

For genomic southern blots, 30 µg of DNA from the T12 transgenic maize leaves was digested with *Hin*d III and *Eco*R I respectively. The digested DNA products were separated on a 1% agarose gel, transferred to a nylon membrane and hybridised with a ^32^P-labeled random primed fragments generated from the 681 bp *rnc*70 gene.

### Purification of His-tagged RNC70 Proteins and Antibody Preparation

The *rnc*70 gene was amplified with the RNC70-1 (5′-CGCGGATCCATGAACCCCATCGTAATTAATC-3′) and RNC70-2 (5′-CCGCTCGAGTCATTCCAGCTCCAGTTTTTTC-3′) primers. The *Bam*H I and *Xho* I sites (underlined), of the primers were used for introduction into the prokaryotic expression vector pET-30a(+). The recombinant vector was then transformed into *E. coli* strain BL21. One liter bacterial cultures were grown to 0.6 OD_600_ from selected colonies, induced with 1 mM IPTG, and shaken overnight at 28°C. The induced cells were lysed by sonication and centrifuged at 4°C, 12000 rpm for 30 min. The cleared lysates were passed through nickel resin (Qiagen China Co., Shanghai) and the His-tagged fusion proteins were eluted with 100 mM imidazole and used to elicit antibodies and for dsRNA binding. Fusion proteins used to elicit rabbit antibodies were dialysed in 50 mM PBS buffer with 500 mM NaCl before intramuscular injections. Fusion proteins used for dsRNA binding assays were dialysed against 20 mM Tris-HCl (pH 6.4), 50 mM NaCl, 1 mM EDTA, 0.5 mM DTT and 50% glycerol [72].

### Western Blot and ELISA Assays

For western blots, 0.1 g of fresh leaf tissues were collected and soluble proteins were extracted by grinding in 200 µl of protein loading buffer, separated on 15% SDS-PAGE and transferred to nitrocellulose filters. Primary antibodies against RNC70 were used at a dilution of 1∶500, and the enzyme-linked secondary goat anti-rabbit antibody conjugated to alkaline phosphatase (AP-A) (Sigma Aldrich, St. Louis, MO) was diluted to 1∶5000. The AP-A substrate, BCIP/NBT, was added, and the reaction was stopped by washing out the substrate.

For ELISA, 50 mg of fresh maize leaves were extracted in 200 µl of 10 mM ammonium citrate buffer (pH 6.5) for evaluation of total protein. Duplicate samples consisting of 80 µl of extraction solution and 20 µl of citrate buffer were added to 96 well plates, and incubated overnight at 4°C. The plates were washed with 0.02 M phosphate-buffered saline containing 0.05% Tween 20 (PBST) and then blocked with 3% BSA prepared in PBST for 2 h in 37°C. After PBST washing, plates coated with bound antibodies against the outer capsid (P10) of RBSDV for 2 h in 37°C [73]. Then, the PBST sample washing was repeated, and the secondary AP-A antibody was added to the plates, incubated for 1 h in 37°C. In the final step, plates were rinsed with PBST, incubated with p*-nitrophenyl-phosphate* (*pNPP)*, and the OD_405_ was determined with a spectrophotometer (Model 650, Bio-Rad).

### Experimental Transmission of RBSDV to Maize by the Small Brown Planthopper

Healthy *L. striatellus* second instar nymphs were placed on wheat infected with RBSDV for 3 days of virus acquisition and feeding, followed by transferring to healthy wheat. After 20 days, the number of planthoppers containing RBSDV was assessed by ELISA and shown to range from 23 to 27%. Then, 800 planthoppers were transferred to 210 maize seedings at 10 days after planting, when the first two true leaves had fully expanded and the leaf whorl was beginning to emerge. The plants were placed in an insect-proof net cage (60 mesh), and after a 3-day inoculation access period, the planthoppers were killed with an insecticide. The maize plants were grown to the four-leaf stage, and transplanted to an experimental plot covered with a fly net to prevent external planthopper access. Leaves were collected for molecular detection after 10 to 15 days when visible symptoms appeared, and the plants were grown to maturity to assess the disease indices.

### RBSDV dsRNA Genome Isolation and Binding with RNC70

To isolate dsRNA, 5 g of fresh RBSDV infected maize leaves were ground in liquid nitrogen, and 9 ml of 1× STE (0.05 M Tris-HCl (pH 6.8), 0.1 M NaCl, 1 mM EDTA) and 1 ml 2% SDS was added. An equal volume of water saturated phenol: chloroform: isoamylalcohol (25∶24∶1) was added and stirred with a magnetic stirrer for 30 min at room temperature. The samples were centrifuged at 8000 rpm for 20 min, the supernatant was recovered, and ethanol was added to a final concentration of 16%. The mixture was then passed through a 2.5 g cellulose CF11 affinity column and washed with 30 ml of 1× STE containing 16% ethanol followed by a second wash with 120 ml of 1× STE containing 16% ethanol to remove residual ssRNA. The dsRNA was eluted with 15 ml of 1× STE without alcohol and the eluent was collected. RNA was precipitated by adding 1/10 volume of 3 M NaAc and 2.5 volumes of cold absolute ethanol, and the samples were stored at −20°C for at least 2 h. Samples were then centrifuged at 12000 rpm for 15 min washed twice with 70% alcohol and the pellets were dissolved in ddH_2_O or 1× STE and stored at −20°C.

RNA binding was carried out in reactions containing 75 ng of purified RBSDV genomic dsRNA and 0.11, 0.22 or 0.33 µg of his-tagged RNC70 protein in 10 mM Tris-HCl, 10 mM MgCl_2_, 50 mM NaCl, 1 mM dithiothreitol (pH 7.9). The reactants were incubated for 30 min at 37°C, and electrophoresed on 5% native polyacrylamide gels at 20 V for 4 h before ethidium bromide staining and photography of the gels.

### Resistance Evaluation and Statistical Analysis of Transgenic Maize

To evaluate the resistance of transgenic maize plants under field conditions, transgenic plants and non-transgenic controls were grown in areas with the highest disease incidences in the region (Figure 3). Depending on the availability of planting material, two to ten replicates of single-row plots per transgenic line were grown using a randomized block design. Each row, consisting of ten to twenty plants, was planted with seed from a randomized selected ear of each transgenic maize line.

Every maize plant evaluated was assigned a disease score and the numbers of plants corresponded to the numbers of plant lines recommended by the SPSS analysis package described below. All infectivity data were incorporated into Microsoft Excel 2010, and the results were analyzed with SPSS Statistics software (version 17, IBM). The statistical analyses were calculated using the disease scores and the corresponding numbers of plants in each line were analyzed by Duncan’s multiple range tests to evaluate statistical differences between the resistances of each line.
