# Correction: Enhanced Virus Resistance in Transgenic Maize Expressing a dsRNA-Specific Endoribonuclease Gene from *E. coli*

**DOI:** 10.1371/annotation/94a7b65a-b630-4875-bb2d-917a4194c3bb

**Published:** 2013-10-21

**Authors:** Xiuling Cao, Yingui Lu, Dianping Di, Zhiyan Zhang, He Liu, Lanzhi Tian, Aihong Zhang, Yanjing Zhang, Lindan Shi, Bihong Guo, Jin Xu, Xifei Duan, Xianbing Wang, Chenggui Han, Hongqin Miao, Jialin Yu, Dawei Li

There were multiple errors in the Author Contributions statement. The second author (YL) was incorrectly included in the list of authors who "Wrote the paper." In addition, the first author (XC) should be included in the list of authors who "Wrote the paper." 

